# A Comprehensive LOVD Database for Fatty Acid Oxidation Disorders in Chinese Populations

**DOI:** 10.1155/2023/5493978

**Published:** 2023-07-25

**Authors:** Ting Zhang, Zinan Yu, Lingwei Hu, Chao Zhang, Haixia Miao, Rulai Yang, Ming Qi, Benqing Wu, Xinwen Huang

**Affiliations:** ^1^Department of Genetics and Metabolism, Children's Hospital of Zhejiang University School of Medicine, National Clinical Research Center for Child Health, Hangzhou, China; ^2^Assisted Reproduction Unit, Department of Obstetrics and Gynecology, Sir Run Run Shaw Hospital, Zhejiang University School of Medicine, Hangzhou, Zhejiang, China; ^3^DIAN Diagnostics, Hangzhou, Zhejiang, China; ^4^Department of Pathology and Laboratory Medicine, University of Rochester Medical Center, Rochester, New York, USA; ^5^Department of Neonatology, Children's Medical Center, University of Chinese Academy of Science, Shenzhen Hospital, Shenzhen, Guangdong, China

## Abstract

Fatty acid oxidation disorders (FAODs) are a group of rare, autosomal recessive, metabolic disorders with clinical symptoms from mild types of fatigue, muscle weakness to severe types of hypoketotic hypoglycemia, (cardio)myopathy, arrhythmia, and rhabdomyolysis, especially during prolonged fasting, exercise, and illness. There are eleven diseases caused by thirteen FAOD genes (*SLC22A5*, *ETFDH*, *ETFA*, *ETFB*, *SLC25A20*, *ACADS*, *ACADM*, *ACADVL*, *ACAT1*, *CPT1A*, *CPT2*, *HADHA*, and *HADHB*) which are specific enzymes or transport proteins involved in the mitochondrial catabolism of fatty acids. We built the LOVD database for FAODs focused on the Chinese population, in which we recorded all the reported variants by literature peer review. In addition, the unpublished variant data of patients from Zhejiang province were also incorporated into the database. Currently, a total of 538 unique variants have been recorded. We also compared the incidence of high-frequency variants of certain FAOD genes among different populations. The database would provide the guidance for genetic screening of Chinese patients.

## 1. Introduction

The mitochondrial fatty acid oxidation (FAO) is critical to the supply of ATP in tissues with high energy consumption, including the heart, skeletal muscle, and liver. Fatty acid oxidation disorders (FAODs) are a group of rare, autosomal recessive, metabolic disorders caused by defects of mitochondrial catabolism of fatty acids, resulting in accumulation of characteristic fatty acids and carnitine derivatives. Diagnosis is made through tandem mass spectrometry based on the acylcarnitine profiling of dried blood spots in the newborn screening. The combined incidence of FAODs from Australia, Germany, and the USA is approximately 1 : 9,300 [1], while the incidence is much lower in Asia such as Japan (1 : 30,000), South Korea (1 : 111,000) [2], and China (1 : 15,382) [3]. Most FAOD patients diagnosed by newborn screening have no clinical symptoms but with elevation of acylcarnitines. Some patients developed clinical symptoms from mild types with fatigue, muscle weakness to severe types with acute metabolic decompensation, hypoketotic hypoglycemia, cardiomyopathy, hepatopathy, recurrent rhabdomyolysis, and encephalopathy, especially during prolonged fasting, exercise, and illness. Early diagnosis and timely treatment can significantly improve their prognosis.

**Figure 1:**
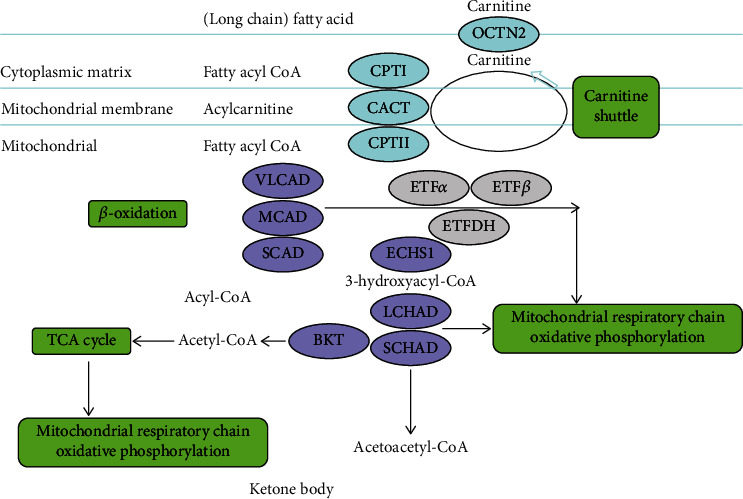
Mechanism of fatty acid mitochondrial catabolism.

There are eleven diseases caused by the lack of specific enzymes or transport proteins involved in the mitochondrial catabolism of fatty acids. In the cytosol, fatty acids are activated to acyl-coenzyme A (CoA) esters before they can be directed into different metabolic pathways. The mitochondrial import of acyl-CoAs requires the carnitine cycle which is composed of L-carnitine and two acyltransferases, carnitine palmitoyltransferases 1 and 2 (CPT1 and CPT2), and carnitine acyl-carnitine translocase (CACT) (Figure 1). Deficiency of this system leads to primary carnitine deficiency (PCD), carnitine-acylcarnitine translocase deficiency (CACTD), carnitine palmitoyltransferase 1 deficiency (CPT1D), and carnitine palmitoyltransferase 2 deficiency (CPT2D) [4]. Inside the mitochondrion, acyl-CoAs are degraded by *β*-oxidation cycle consisting of four enzymatic steps (Figure 1). Each cycle shortens acyl-CoA by the successive removal of two carbon fragments [5]. Deficiency of this cycle leads to short-chain acyl-CoA dehydrogenase deficiency (SCADD), medium-chain acyl-CoA dehydrogenase deficiency (MCADD), very-long-chain acyl-CoA dehydrogenase deficiency (VLCADD), and mitochondrial trifunctional protein (MTP) deficiency. The ETF/ETFDH complex transfers electrons from the dehydrogenases to the electron transport chain, and the deficiency leads to multiple acyl-CoA dehydrogenase deficiency (MADD). Furthermore, the *β*-ketothiolase deficiency (BKTD) is caused by defects in the metabolism of extrahepatic ketone bodies and the pathway of isoleucine catabolism, which contribute to the last step of fatty acid oxidation.

Since false positive results accounted for a fraction of recalled newborns due to influence of maternal acylcarnitine levels or the factors in the detection, the genetic testing is golden standard of diagnosis for the diseases. To date, hundreds of variants have been found in these FAOD-associated genes. However, discerning the clinical relevance and the pathogenicity of variants is still a challenge. Variant databases are essential for both researchers and clinicians to improve the knowledge of the diseases. Although there are some variant databases for each FAOD gene in Leiden Open Variation Database (LOVD), ClinVar, and Human Gene Mutation Database (HGMD), most of the data were collected from Caucasian and Ashkenazi Jewish populations. The comprehensive Chinese-specific variant databases for FAOD genes are still lacking. To address this gap, we present a comprehensive variant database of FAODs, focused on the Chinese population, recording the details of all the reported variants through literatures, as well as unpublished data from our laboratory. The database includes 538 variants in total for 13 disease genes, of which 328 variants in our laboratory and the majority of variants in literatures are found through newborn screening. We also compared the incidence of high-frequency variants of certain FAOD genes among different populations. Thus, the database would provide the guidance for genetic screening of Chinese patients.

## 2. Methods and Results

### 2.1. LOVD of FAOD Variants in a Chinese Population

#### 2.1.1. Data Collection and Database Content

The variant data was collected from PubMed (https://www.ncbi.nlm.nih.gov/pubmed) and Chinese core journals (http://www.wanfangdata.com.cn/;http://mqikan.cqvip.com), as well as the unpublished data derived from high-throughput sequencing data of patients of FAODs in Zhejiang province from our laboratory. The variants are verified by Mutalyzer (https://mutalyzer.nl/). The study was approved by the Ethical Committee of Children's Hospital, Zhejiang University School of Medicine (reference number: 2020-IRBAL-035). A total of 538 unique variants in 13 FAOD genes (*SLC22A5*, MIM# 603377; *ETFDH*, MIM# 231675; *ETFA*, MIM# 608053; *ETFB*, MIM# 130410; *SLC25A20*, MIM# 613698; *ACADS*, MIM# 606885; *ACADM*, MIM# 607008; *ACADVL*, MIM# 609575; *ACAT1*, MIM# 607809; *CPT1A*, MIM# 600528; *CPT2*, MIM# 600650; *HADHA*, MIM# 600890; and *HADHB*, MIM# 143450) are recorded (Table 1). The corresponding data was displayed in http://www.genomed.zju.edu.cn/LOVD3/genes.

#### 2.1.2. Database Structure

**Figure 2:**
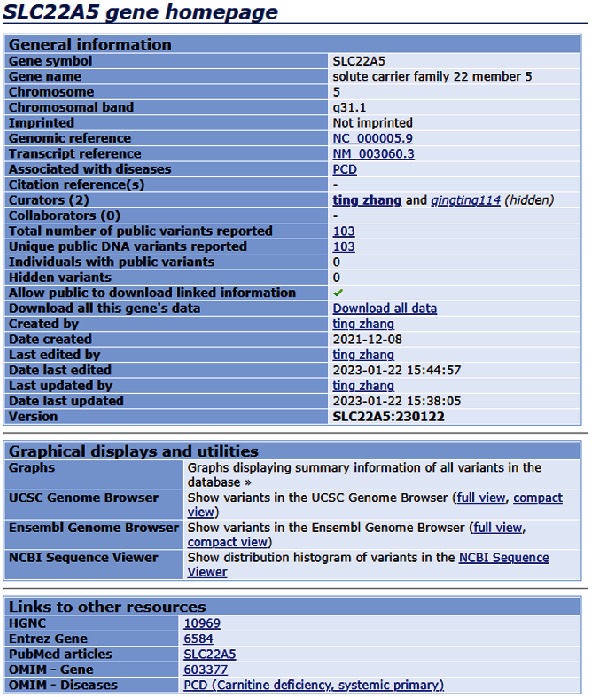
Homepage of the *SLC22A5* gene from our LOVD-China database for FAODs.

The database is a simple table, and the left row shows the thirteen FAOD genes. Each gene links to its own home database. For each variant, the exon, transcript ID, nucleotide change, protein change, frequency in patients, ACMG classification, and cited references are listed. Taking *SLC22A5* as an example (Figure 2), the homepage of the variant database contains the basic information about the *SLC22A5* gene in the general information section, and links to other authoritative resources including Entrez gene, PubMed articles, and Online Mendelian Inheritance in Man in the linkage section. At the top of the web page are function buttons named “Genes,” “Transcripts,” “Variants,” “Individuals,” “Diseases,” “Screenings,” “Submit,” and “Documentation.” The remote user can search the data and is encouraged to submit new variants after registering as a submitter.

#### 2.1.3. Data Submission

The LOVD-China database is available for public submission. Submitters should complete the variant data and other information in detail. The authors of this study are responsible for the control of each entry, adding new entries, and updating existing variant data. More detailed information can be found at http://www.genomed.zju.edu.cn/LOVD3/docs/.

## 3. Discussion

The LOVD-China database was firstly built by Zhejiang University as part of the International Human Variome Project. It has already recorded comprehensive phenotype-genotype datasets from China, including breast cancer [6], colorectal cancer, long QT syndromes (LQTS) [7], and hemoglobinopathies [8]. We integrated all the collectable variants of FAODs in the Chinese population to establish the LOVD-China database. The purpose of this study is to display a comprehensive variation spectrum of FAODs on the Chinese population. The database will not only assist clinical geneticists in interpreting the genetic variation of these genes but also aid genetic scientists in investigating the function of the variants. By comparing and analyzing the variation spectrum among different populations, we can improve the knowledge of the ethnic-specific molecular characteristics in different populations of certain diseases.

### 3.1. Carnitine Cycle Defects

**Figure 3:**
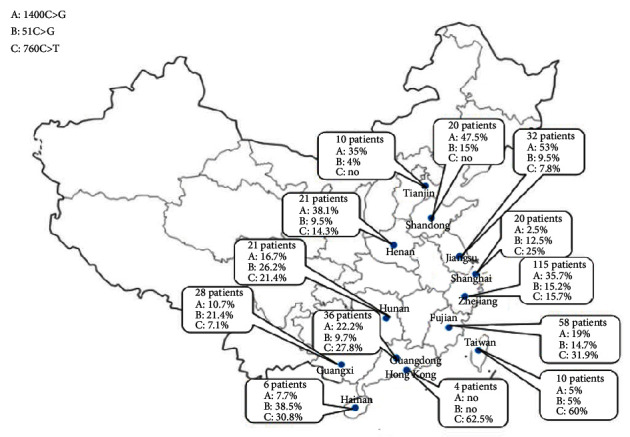
The geographical distribution of three high-frequency variants of *SLC22A5* in China.

The primary carnitine deficiency (PCD) is caused by defect of the organic cation transporter OCTN2 on the cell membrane, which is encoded by *SLC22A5* gene and transports carnitine across the plasma membrane. To date, more than 150 disease-causing variants have been reported worldwide [9], and our database recorded 103 variants occurred in the Chinese population. The high-frequency variants, namely, c.1400C>G (p.S467C), c.51C>G (p.F17L), c.760C>T (p.R254X), c.338G>A (p.C113Y), and c.428C>T (p.P143L), account for 33.1%, 18.3%, 7.3%, 4.8%, and 4.8%, respectively. By analysis of the geographical distribution, c.1400C>G (p.S467C) is the most common variant in Henan, Tianjin, Shandong, Jiangsu, and Zhejiang provinces, while c.51C>G (p.F17L) is most common in Guangxi, Hunan, and Hainan provinces (Figure 3). c.760C>T (p.R254X) is the most prevalent variant in southern part of China including Fujian, Shanghai, Guangdong, Hong Kong, and Taiwan. However, it is rarely reported in western countries. The previous studies have revealed founder variants in several populations, such as c.396G>A (p.W132X) and c.1400C>G (p.S467C) in Japan [10] as well as c.95A>G (p.N32S) in the Faroe Islands [11]. However, c.396G>A (p.W132X) is only found in one case, and c.95A>G (p.N32S) accounts for 0.75% in our database. Moreover, in the United States, c.136C>T (p.P46S) is the most common variant [9], while none has been found in China yet.

The carnitine palmitoyltransferase 1 deficiency (CPT1D) is mainly caused by variants in *CPT1A* gene which encodes CPT1A, an integral outer mitochondrial membrane protein catalyzing the transesterification of the acyl-CoA to acylcarnitine. Reports on Chinese patients with *CPT1A* deficiency are limited. Only 28 different variants of *CPT1A* in Chinese patients are identified in our database. c.2201T>C (p.F734S) in exon 18 was the most frequent variant, accounting for 11.1%. This variant has not yet been found in other populations and could be a unique high-frequency variant of Chinese populations. In the USA, c.1436C>T is the most prevalent variant that up to 80% of native infants are homozygous for the c.1436C>T in Alaska, but it has not been reported in China [12].

The carnitine palmitoyltransferase 2 deficiency (CPT2D) is caused by lack of CPT2 which reconverts the acylcarnitine into an acyl-CoA inside the mitochondria and is encoded by *CPT2*. 16 variants of *CPT2* in Chinese patients are identified in our database. c.1711C>A (p.P571T) was the most frequent variant, accounting for 25%, followed by c.1055T>G (p.F352C) and c.1102G>A (p.V368I), which accounted for 15.6% and 12.5%, respectively. It is reported that patients harboring either c.1055T>G (p.F352C) or c.1102G>A (p.V368I) are prone to influenza-associated encephalopathy (IAE). These two variants are much more prevalent in the Japanese and Chinese populations but have not been reported in Caucasians [13].

The carnitine-acylcarnitine translocase deficiency (CACTD) is caused by defect of CACT which catalyzes acylcarnitines to transport across the inner mitochondrial membrane in exchange of a free carnitine molecule. It is caused by variants of *SLC25A20* consisting of 9 exons. At present, there are 10 variants of *SLC25A20* gene in our database. Aberrant mRNA splicing appears to be a relatively common phenomenon in *SLC25A20* gene. c.199-10T>G in intron 2 is the most common pathogenic variant in China, which accounts for 77.9%. This variant occurred mostly often in Asia such as China, Vietnam, Japan, and Thailand [14]. Hsu et al. found that c.199-10T>G resulted in the omission of exon 3 or exon 3+4 and truncation of CACT enzyme protein, which would lead to poor outcome and high mortality [15].

### 3.2. The *β*-Oxidation Cycle Defects

In humans, three different acyl-CoA dehydrogenases, the very-long-chain, medium-chain, and short-chain acyl-CoA dehydrogenases (VLCAD, MCAD, and SCAD) carry out the metabolism of acyl-CoAs from long- to medium- and eventually to short-chain acyl-CoAs.

The short-chain acyl-CoA dehydrogenase deficiency (SCADD) is caused by variants of *ACADS* gene. More than 70 *ACADS* variants have been reported worldwide, and most are missense variants. 38 variants of *ACADS* in Chinese patients are recorded in our database. It appears that c.1031A>G (p.E344G) in exon 9 and c.164C>T (p.P55L) in exon 2 have the highest detection rates in Chinese patients, accounting for 34.6% and 15.6%, respectively. In the American [16], European, and Jewish [17] populations, c.625G>A (p.G209S) and c.511C>T (p.R171W) are the most common variants. The frequency of c.625G>A (p.G209S) was 30% in patients with Spanish origin, 35% in Germany, 40% in the Netherlands, and 22% in the United States [17], but only 3.9% in our database. The variation frequency of c.511C>T (p.R171W) was 8% in Western Europe and 3% in the United States [18], respectively, while it has not been reported in China yet.

The medium-chain acyl-CoA dehydrogenase deficiency (MCADD) is caused by variants in *ACADM* gene. There are 56 variants of *ACADM* gene in our database. c.449_452del (p. T150Rfs∗4) in exon 6 is the most common variant in East Asian patients [19], including Japanese [20], South Koreans [21], and Chinese (showed in our database). However, c.985A>G (p.K329E) occurs most frequently in Caucasian patients of Northern European descent [22], which is not yet detected in China.

The very-long-chain acyl-CoA dehydrogenase deficiency (VLCADD) is caused by variants of *ACADVL*. So far, about 260 variants have been reported worldwide. There are 93 variants of *ACADVL* gene in our database. c.1349G>A (p.R450H) in exon 14 is the most common variant in Chinese patients, accounting for 12.8%. Two variants, c.664G>A and c.664G>C, leading to the same amino acid change (p.G222R), account for 2.1% and 4.3%, respectively, in our database and might only be reported in people with Chinese origin.

**Figure 4:**
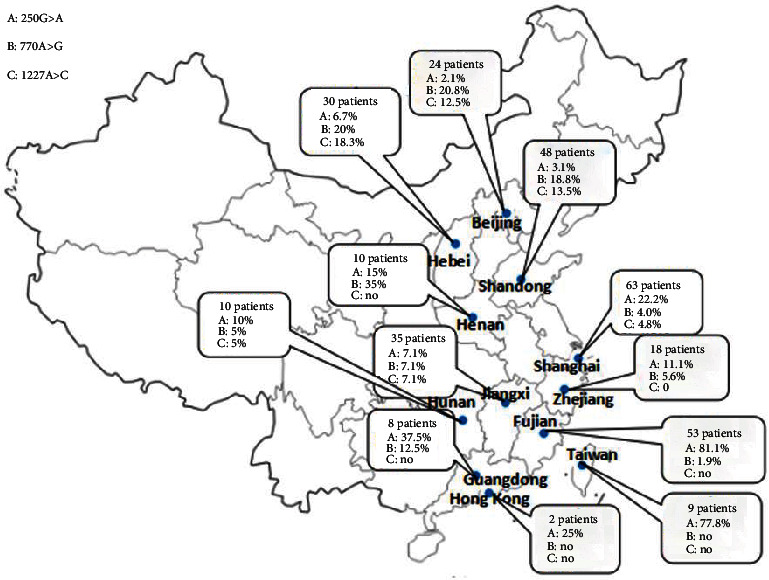
The geographical distribution of three high-frequency variants of *ETFDH* in China.

The multiple acyl-CoA dehydrogenase deficiency (MADD), also called GAII, is caused by genetic defects in the electron transfer flavoprotein ETF and its dehydrogenase ETFDH, which are encoded by *ETFA*, *ETFB*, and *ETFDH*, respectively. There are 143 *ETFDH* variants identified in our database. c.250G>A (p.A84T), c.770A>G (p.Y257C), c.1227A>C (p.L409F), and c.389A>T (p.L127P) account for 29.8%, 10.4%, 6.6%, and 4.9%, respectively (Figure 4). c.250G>A (p.A84T) is the most common *ETFDH* pathogenic variant in the southeast of China, including in Hunan, Shanghai, Zhejiang, Guangdong, and Hong Kong, especially in Fujian and Taiwan [23]. It is also most prevalent in Southeast Asia because of the migration and distribution of Southern Min population in Southern China and neighboring countries [24]. However, the c.250G>A (p.A84T) variant is not yet reported in western countries. c.770A>G (p.Y257C) is the most common variant in Northern China, including Beijing, Shandong, Hebei, and Henan provinces. These results further confirmed that there are different founder variants of *ETFDH* variants between the southeastern and northern Chinese populations [25]. Fu et al. [26] found that patients with severe metabolic symptoms often presented with a wide spectrum of *ETFDH* gene variants without high-frequency variants. Reports on the Chinese patients with *ETFA* and *ETFB* variants are limited; only 6 variants of *ETFA* and 2 variants of *ETFB* are recorded in our database.

The mitochondrial trifunctional protein (MTP) is a multienzyme complex that catalyzes the last three steps of long-chain mitochondrial fatty acid *β*-oxidation. It consists of four *α* and four *β* subunits encoded by the genes *HADHA* and *HADHB*, respectively. In the Caucasian group, the variation frequency of *HADHA* and *HADHB* is similar [27, 28]. However, in Asian patients, 80% patients harbor *HADHB* variants [29]. Only two variants of *HADHA* gene from one patient and fourteen variants of *HADHB* gene are recorded in our database. c.739C>T (p.R247C) in exon 9 and c.1175C>T (p.A392V) in exon 14 are the rather common variants in *HADHB*, accounting for 19.2% and 15.4%, respectively.

### 3.3. The *β*-Ketothiolase Deficiency (BKTD)

The BKTD is caused by defect in *ACAT1* gene which encodes the acetoacetyl-CoA thiolase (ACAT1). ACAT1 catalyzes the metabolism of extrahepatic ketone bodies and isoleucine and may also contribute to the last step of fatty acid oxidation. At present, there are 28 variants of *ACAT1* gene in our database. c.622C>T (p.R208X) and c.1124A>G (p.N375S) may be the high-frequency variants in general Chinese patients with BKTD, accounting for 16.9% and 8.5%, respectively. The allele frequency of c.622C>T (p.R208X) is especially high in Guangdong and Guangxi of China, and it is also the main variant in Vietnam with a frequency of 87.5% [30, 31]. The clinical manifestations of c.1124A>G (p.N375S) were reported to associate with nervous system damage [32]. In India, c.578T>G (p. M193R) was the main variant with a frequency of 45% [33] but has not been found in China yet.

In conclusion, the novel LOVD-China database for FAODs will provide a great convenience for researchers and clinicians to study, test, and diagnose FAODs caused by variants on the involved genes. The database helps us to improve molecular spectrum in Chinese population and facilitates future genetic tests worldwide. Due to the limitation of data collection or the lack of functional studies at protein level, so far, the connection between genetic variants and clinical manifestations is not very clear yet. In the future, if a new variant is identified, we will update the database. We hope this database will be enriched with the help of remote users and scholars that may submit their own variants.

## Figures and Tables

**Table 1:** Summary of variants of FAODs in LOVD-China.

Gene	*SLC22A5*	*ETFDH*	*ETFA*	*ETFB*	*ACADS*	*ACADM*	*ACADVL*	*SLC25A20*	*ACAT1*	*CPT1A*	*CPT2*	*HADHA*	*HADHB*
OMIM	603377	231675	608053	130410	606885	607008	609575	613698	607809	600528	600650	600890	143450
Location	5q31.1	4q32.1	15q24.2-q24.3	19q13.41	12q24.31	1p31.1	17p13.1	3p21.31	11q22.3	11q13.3	1p32.3	2p23.3	2p23.3
Total variants^∗^	103	143	6	2	38	56	93	10	28	28	15	2	14

^∗^The total numbers of variants occurred in the patients of each gene.

## Data Availability

The variant data was collected from PubMed (https://www.ncbi.nlm.nih.gov/pubmed) and Chinese core journals (http://www.wanfangdata.com.cn/;http://mqikan.cqvip.com) and also the unpublished data derived from high-throughput sequencing data of patients of FAODs in Zhejiang province from our laboratory. The mutations are verified by Mutalyzer (https://mutalyzer.nl/). The corresponding data was displayed in http://www.genomed.zju.edu.cn/LOVD3/genes.
